# Effect of incorporation of triphala into AH26 sealer on its cytotoxicity at different intervals

**DOI:** 10.34172/joddd.2020.035

**Published:** 2020-09-21

**Authors:** Mahsa Eskandarinezhad, Amir Hooman Sadr Haghighi, Sahar Khademnezhad, Zahra Aghazadeh, Fateme Noruzani

**Affiliations:** ^1^Department of Endodontics, Faculty of Dentistry, Tabriz University of Medical Sciences, Tabriz, Iran; ^2^Department of Orthodontics, Faculty of Dentistry, Tabriz University of Medical Sciences, Tabriz, Iran; ^3^Department of Oral Medicine, Faculty of Dentistry, Tabriz University of Medical Sciences, Tabriz,Iran; ^4^Undergraduate Student, Faculty of Dentistry, Tabriz University of Medical Sciences, Tabriz, Iran

**Keywords:** AH26, Gingival fibroblasts, Triphala

## Abstract

**Background.** One of the essential properties of sealers used during endodontic treatment is their biocompatibility. Different materials are added to these sealers to improve their properties, including antibacterial activity. In recent years, there has been an increase in interest in the use of herbal medicines. This study aimed to evaluate the effect of incorporating triphala into AH26 sealer on its cytotoxicity on gingival fibroblasts at different intervals after mixing.

**Methods.** In the present in vitro study, the cytotoxicity of AH26 sealer was evaluated once in its pure form and once after mixing it with triphala at 24-, 48-, and 72-hour, and 7-day intervals after mixing using the standard MTT assay protocol on gingival fibroblasts.

**Results.** Two-way ANOVA was used to evaluate the effect of groups on the mean changes in cytotoxicity at different time intervals at a significance level of P<0.05. The results showed that the incorporation of triphala into the AH26 sealer did not increase or decrease its cytotoxicity (P=0.909). Besides, there was a decrease in cytotoxicity in both study groups. However, there was a relative increase in the sealers’ cytotoxicity in both groups in the first 72 hours (P<0.0001).

**Conclusion.** Considering the well-established antibacterial properties of triphala in our previous study, the present study’s results showed that the incorporation of triphala into the AH26 sealer did not increase the cytotoxicity of the sealer. Therefore, it can be incorporated into the AH26 sealer to improve the other properties of the sealer, including its antibacterial activity.

## Introduction

The most commonly used root canal filling materials are gutta-percha and endodontic sealers. It is necessary to use sealers during the obturation of root canals for the success of endodontic treatment. Sealers fill the tiny gaps between the primary root canal filling material and the root canal wall and serve as a lubricating material for sliding and easier placement of the primary root canal obturation material. In addition, they fill the accessory root canals and porosities along the root canal.^1^ According to Grossman, the ideal properties of a root canal filling material include tissue compatibility, absence of any shrinkage during setting, long setting time, adhesion, radiopacity, no effect on tooth discoloration, solubility in a solvent, insolubility in the oral and tissue fluids, induction of a seal, and antimicrobial activity.^2^ The sealers used in endodontic treatment and its by-products within the root canal are at close proximity to the extracellular fluid and periradicular tissues. Therefore, they can cause different reactions around the root. Cytotoxic agents, too, can cause inflammatory responses and tissue damage. As a result, one of the essential characteristics of root canal sealers is their biocompatibility.^3^

At present, a large proportion of sealers used in the endodontic treatment are resin-based sealers, including AH26,^4^ which is a very useful and commonly used sealer; however, it is cytotoxic in root canal therapy. The cytotoxic effect is due to the release of small amounts of formaldehyde during its chemical setting.^5^ After 24 hours, its toxicity reaches a minimum in vivo and in vitro.^6^

Currently, different materials, including antibiotics, are incorporated into sealers to increase their antibacterial activity. However, considering the side effects of different medications and an increase in bacterial resistance to these medications, there has been an increasing interest in the use of herbal medicines.

The essential advantages of herbal medicines include their ready availability, low cost, long shelf-life, low toxicity, and the absence of microbial resistance.^7^ One of these medications, which has recently been used in dentistry, is triphala, an Indian herbal medicine. Triphala means three (tri) fruits (phala). Its powder consists of a combination of three dried herbs (*Terminalia bellirica*, *Emblica officinalis* , and *Terminalia chebula*). This herbal medicine is used to treat conditions such as headaches, constipation, and hepatic disorders in traditional Indian medicine.^8,9^ This material has considerable dental applications due to its antimicrobial effects. Oral rinse containing triphala is used in controlling tooth decay and in periodontal therapies. Studies have shown it can be used as a root canal irrigant due to its antibacterial and antioxidant characteristics.^10^ In our previous study, we compared the antimicrobial activity of triphala with different concentrations of sodium hypochlorite (NaOCl) against *Enterococcus faecalis* (*E. faecalis*). Our experiment showed significant antimicrobial effects of triphala.^11^

Since knowledge about the biocompatibility of sealers is essential for their use in the treatment procedures and since no data are available on the cytotoxic effects of AH26 sealer mixed with triphala in the long term after mixing, this study was undertaken to evaluate the effect of incorporation of triphala into AH26 sealer on its cytotoxicity to gingival fibroblasts at different intervals after mixing.

## Methods

This in vitro experiment was conducted in the Faculty of Dentistry, Tabriz University of Medical Sciences, under the ethics code of IR.TBZMED.REC.1396.412.

Human gingival fibroblast cell line (HFF2) was procured from the Pasteur Institute in Tehran and cultured within flasks on DMEM + 10% FBS + 1×Ab (pen/strep) medium, followed by incubation at 37ºC and 5% CO_2_ for growth and proliferation. The culture medium was refreshed every 72 hours. After the 75-mL flask was filled, the cells were counted using a Neubauer microscope plate and trypan blue staining under a light microscope. A Neubauer plate is a thin glass microscope plate consisting of nine squares measuring 1×1 mm; 15–20 µL of the cellular suspension was stained by trypan blue and placed on the plate with the use of a p-20 Pipetman and covered with the glass cover. The aim was to have 100–200 cells in each square. The plate was fixed so that the cells would be immobilized. Then the cells were counted under a light microscope.

### Evaluation of Cytotoxicity (MTT Assay)

**Figure 1:**
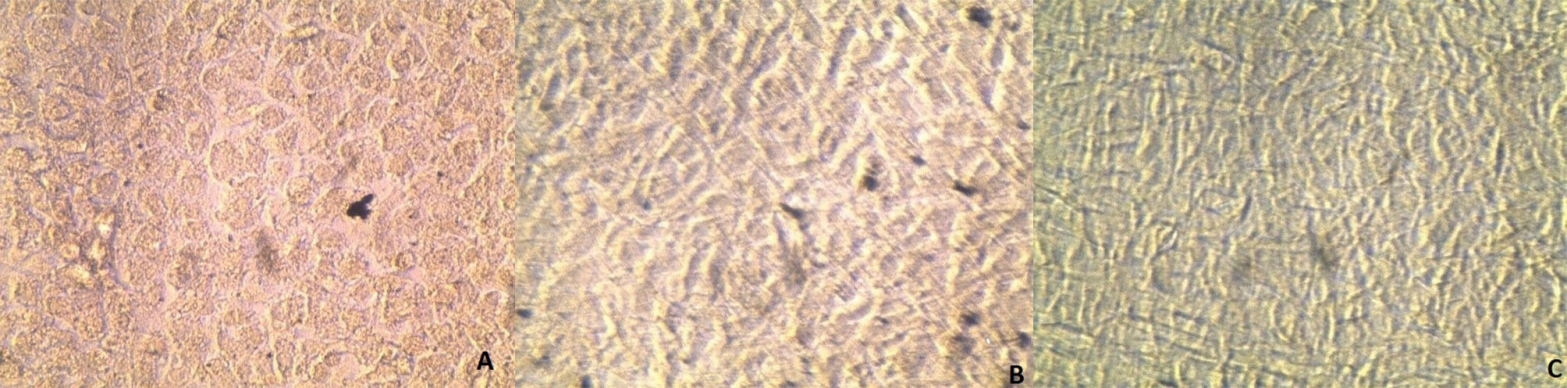
The microphotographs of the cells stained with the MTT assay in the study groups at the 24-hour interval. A: AH26 sealer; B: AH26 sealer + triphala; C: the control groups (×400).

**Figure 2:**
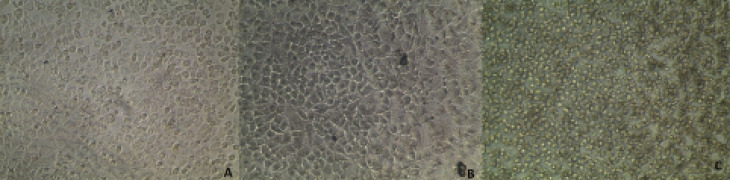
The microphotographs of the cells stained with the MTT assay in the study groups at the 7-day interval. A: AH26 sealer; B: AH26 sealer + triphala; C: the control groups (×400).

Four 96-well plates were prepared. Eight wells were assigned to pure AH26 sealer, eight wells to AH26 sealer mixed with triphala, and eight wells to the control group (without a sealer and only fibroblasts). To evaluate the cytotoxic effect of the sealers, pure AH26 sealer (DENTSPLY) and AH26 sealer mixed with triphala at 10 wt% (IMPCOPS Ltd, Chennai, India) were prepared as a resultant paste and then placed at the floors of the relevant wells with the use of a 40-µm mesh. Then, the gingival fibroblasts were trypsinized and inoculated into the relevant wells at a concentration of 5000 cells/200 µL of the culture medium. After 24 hours, 30 µL of the MTT solution (SIGMA 11465007001) was added to each well. This solution was prepared at a concentration of 5 mg/µL of powder in sterile PBS. After four hours of incubation at 37ºC, 180 µL of the supernatant was removed, and 150 µL of DMSO (dimethyl sulfoxide) was added, followed by 20 minutes of shaking on a shaker. Then, the optical density (OD) of the solution was read by an ELISA Reader at 570- and 490-nm wavelengths.^12^ The same procedures were repeated at 48- and 72-hour and 7-day intervals (Figure 1, 2).

It should be emphasized that eight wells were used in the present study, based on similar studies, to increase the accuracy of the study and the statistical analyses.^13,14^

### Statistical Analysis

The data collected from the ELISA Reader were analyzed with two-way ANOVA, using SPSS 17. Statistical significance was set at P<0.05.

## Results

Evaluation of the cytotoxicity of AH26 sealer in seven days showed that the sealer’s cytotoxicity was high from the beginning to the 72-hour interval, which decreased to a moderate level at the 7-day interval (Table 1).

**Table 1:** The means and severities of the cytotoxicity of AH26 sealer at different time intervals

	**The number of repetitions**	**Mean ± SD**	**Cytotoxicity**
**24 hours**	8	83.68±3.99	high
**48 hours**	8	78.49±4.13	high
**72 hours**	8	86.25±3.02	high
**7 days**	8	53.37±11.98	moderate

Evaluation of the cytotoxicity of AH26 sealer + triphala in seven days showed that the sealer was highly cytotoxic from the beginning to the 72-hour interval, which decreased to a moderate level at the 7-day interval (Table 2).

**Table 2:** The means and severities of the cytotoxicity of AH26 sealer + triphala at different time intervals

	**The number of repetitions**	**Mean ± SD**	**Cytotoxicity**
**24 hours**	8	78.89±7.97	high
**48 hours**	8	77.06±12.18	high
**72 hours**	8	85.26±3.11	high
**7 days**	8	59.74±9.14	moderate

**Figure 3:**
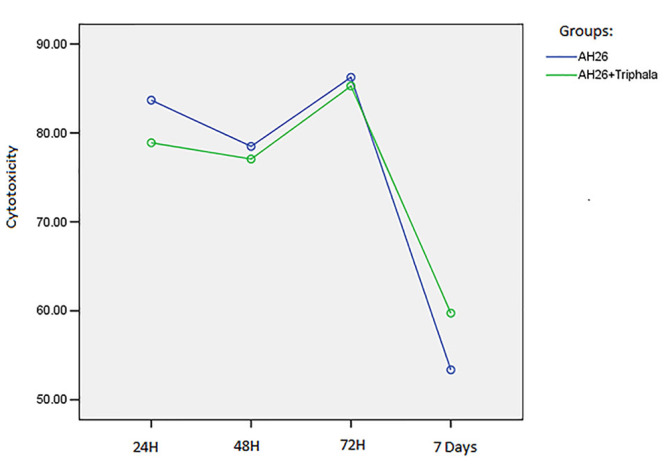
The linear graph of cytotoxicity (%) at different evaluation intervals in the study groups compared to the control group.

Two-way ANOVA was used to evaluate the effect of groups on the mean changes in cytotoxicity at different time intervals at a significance level of P<0.05. The results showed that the incorporation of triphala into the AH26 sealer did not increase or decrease its cytotoxicity (P=0.909). In addition, there was a decrease in cytotoxicity in both study groups. However, there was a relative increase in the sealers’ cytotoxicity in both groups in the first 72 hours (P<0.0001) (Figure 3).

## Discussion

Conventional medications usually provide successful antibiotic therapy for bacterial infections, but they have some side effects. Since resistance to antibacterial agents is very important, in recent years, much attention has been focused on the antimicrobial effects of different herbal drugs. Based on the results of previous studies on the favorable antimicrobial properties of triphala in the dental field, incorporation of triphala into dental sealers might improve their antibacterial properties.,^16^ Since the incorporation of any materials into sealers might affect their other properties, including their cytotoxicity, the present study evaluated this hypothesis. The results showed that the incorporation of triphala into the AH26 sealer did not increase or decrease its cytotoxicity significantly. According to our previous study, triphala has considerable antibacterial effects.^11^ According to our previous study, the antimicrobial effect of triphala was significantly more than NaOCl, which is known as the gold standard of antimicrobial effect in root canal therapy. Therefore, it can be used to improve the antibacterial properties of this sealer.

Triphala has been applied in the treatment of dental and oral diseases. In a clinical trial, the effect of triphala as a mouthwash was compared to chlorhexidine in patients with chronic periodontitis. After 45 days, patients who received triphala mouthwash, in combination with scaling and root planing, exhibited significant reductions in periodontal indices.^17^ The inhibitory effect of triphala on PMN-type matrix metalloproteinase (MMP-9) was assessed in an in vitro study. MMP-9 is expressed in adult periodontitis and plays a pivotal role in the extracellular matrix (ECM) degradation during periodontitis. Triphala significantly decreased the release of MMP-9 in the study.^18^

In a study, the herbal extract of triphala exhibited antioxidant activity and inhibited the dental biofilm formation. Therefore, it was suggested as an effective antiplaque component in toothpastes to protect the gingival cells from free radicals.^19^ Anticariogenic effects of mouthwashes containing triphala were reported in some studies.^20^

In the present study, the cytotoxicity of AH26 sealer during the first 24 hours was high (>70% of cellular death), which decreased significantly over time, except for the 72-hour interval at which a significant increase in cytotoxicity was observed compared to the 48-hour interval. The high cytotoxicity of AH26 might be due to the release of formaldehyde, which is toxic. This data could be attributed to the setting time of the sealers. It is evident that the materials are completely set on the seventh day.

In a study by Huang et al,^3^ the cytotoxicity of AH26 was very high during the first four days, which decreased over time; however, its cytotoxicity was still high.A study by Spangbeng et al^21^ showed that AH26 sealer released a maximum amount of formaldehyde during the first 48 hours after mixing, which decreased after that. Jafari et al^22^ carried out an in vitro study to compare the cytotoxicity of AH26, MTA Fillapex, and Apatite root canal sealers at different time intervals after mixing. They reported that AH26 exhibited high cytotoxicity, which decreased over time.

A study by Javidi et al^23^ showed that the cytotoxicity of AH26 sealer at 1/1, ½ and ¼ dilutions resulted in 90% of cell death during the first 24 hours; however, at 1/8, 1/16, and 1/32 dilutions, the maximum cytotoxicity was observed at 72-hour interval. In another study on human gingival fibroblasts, AH26 sealer exhibited high cytotoxicity immediately after setting, and its cytotoxicity decreased at ¼ and 1/8 dilutions over time, consistent with the results of the present study.^24^ Bakland and Ingle^25^ reported that AH26 is very cytotoxic during the first 24 hours after mixing.Razavian et al^26^ reported a significant difference in the cytotoxicity of freshly mixed and set AH26 sealer. Razmkhah et al^27^ reported that AH26 exhibited moderate cytotoxicity during the 24-hour period after mixing, which persisted up to 48 hours and one week. Shakoei et al^28^ showed that triphala was as effective as 0.5% and 1% NaOCl on *E. faecalis* . Prabhaker et al^7^ showed that NaOCl had the highest antibacterial effect on *E. faecalis* biofilm formed on the dental substrate. Triphala, GTP, and MTAD, too, exhibited significant antibacterial activity.

Pujar et al^15^ compared the effects of triphala, green tea polyphenols and 3% NaOCl on the formation of *E. faecalis* biofilm on the dental substrate in vitro. The results showed that NaOCl had the highest antibacterial activity on the formation of *E. faecalis* biofilm on the dental substrate. GTP and triphala, too, exhibited antibacterial activity against the formation of *E. faecalis* biofilm.

The results of the study above are consistent with those of the present study. Therefore, based on the results of this pioneering in vitro study, triphala has no adverse effects on gingival fibroblasts.

## Conclusion

The results of the present study showed that the incorporation of triphala into AH26 sealer did not increase or decrease the cytotoxicity of this sealer. In our previous study, we evaluated the effective antimicrobial concentration of triphala compared to NaOCl. We added the material in the effective concentration to the AH26 sealer. According to the results, cytotoxicity was the same in both groups. In addition, both groups exhibited a decrease in cytotoxicity over time. Therefore, it is possible to use the favorable properties of triphala, including its antibacterial activity, to improve the sealer’s properties with no concerns about increasing its cytotoxicity.

## Authors’ Contributions

ME was responsible for preparing the sealers, drafting the work or revising it critically for important intellectual content, final approval of the version to be published, and agreement to be accountable for all aspects of the work in ensuring that questions related to the accuracy or integrity of any part of the work are appropriately investigated and resolved. AHSH was responsible for preparing the kits and materials for cell culture, drafting the work or revising it critically for important intellectual content, final approval of the version to be published and agreement to be accountable for all aspects of the work in ensuring that questions related to the accuracy or integrity of any part of the work are appropriately investigated and resolved. SK was responsible for statistical analysis, drafting the work or revising it critically for important intellectual content; final approval of the version to be published; and agreement to be accountable for all aspects of the work in ensuring that questions related to the accuracy or integrity of any part of the work are appropriately investigated and resolved. ZA was responsible for cell culture, drafting the work or revising it critically for important intellectual content, final approval of the version to be published, and agreement to be accountable for all aspects of the work in ensuring that questions related to the accuracy or integrity of any part of the work are appropriately investigated and resolved. FN was responsible for the MTT assay, drafting the work or revising it critically for important intellectual content; final approval of the version to be published, and agreement to be accountable for all aspects of the work in ensuring that questions related to the accuracy or integrity of any part of the work are appropriately investigated and resolved. All the authors have read and agreed to the published version of the manuscript.

## Acknowledgments

None.

## Funding

The authors received no financial support for the research, authorship, or publication of this article.

## Competing Interests

The authors declare no competing interests with regards to the authorship and/or publication of this article.

## Ethics approval

The study protocol was approved by Ethics Committee of Tabriz University of Medical Sciences under the code IR.TBZMED.REC.1396.412.
